# Are smokers rational addicts? Empirical evidence from the Indonesian Family Life Survey

**DOI:** 10.1186/1477-7517-8-6

**Published:** 2011-02-23

**Authors:** Budi Hidayat, Hasbullah Thabrany

**Affiliations:** 1 Department of Health Policy and Administration, Faculty of Public Health, the University of Indonesia, Indonesia; 2 Center for Health Economics and Policy Analyses Studies, Faculty of Public Health, the University of Indonesia, Indonesia

## Abstract

**Background:**

Indonesia is one of the largest consumers of tobacco in the world, however there has been little work done on the economics addiction of tobacco. This study provides an empirical test of a rational addiction (henceforth RA) hypothesis of cigarette demand in Indonesia.

**Methods:**

Four estimators (OLS, 2SLS, GMM, and System-GMM) were explored to test the RA hypothesis. The author adopted several diagnostics tests to select the best estimator to overcome econometric problems faced in presence of the past and future cigarette consumption (suspected endogenous variables). A short-run and long-run price elasticities of cigarettes demand was then calculated. The model was applied to individuals pooled data derived from three-waves a panel of the Indonesian Family Life Survey spanning the period 1993-2000.

**Results:**

The past cigarette consumption coefficients turned out to be a positive with a *p*-value < 1%, implying that cigarettes indeed an addictive goods. The rational addiction hypothesis was rejected in favour of myopic ones. The short-run cigarette price elasticity for male and female was estimated to be-0.38 and -0.57, respectively, and the long-run one was -0.4 and -3.85, respectively.

**Conclusions:**

Health policymakers should redesign current public health campaign against cigarette smoking in the country. Given the demand for cigarettes to be more prices sensitive for the long run (and female) than the short run (and male), an increase in the price of cigarettes could lead to a significant fall in cigarette consumption in the long run rather than as a constant source of government revenue.

## Background

The World Health Organization has developed the WHO Framework Convention on Tobacco Control [1]. This framework is an evidence-based agreement that reaffirms the right of all people to the highest standard of health, and represents a paradigm shift in developing a regulatory strategy to address addictive substances. The key tobacco control policies as reflected in the WHO Framework Convention on Tobacco Control are based on supply and demand reduction strategies. The demand reduction provisions include two main approaches: price and tax measures and non-price measures.

Increasing cigarette prices via excise taxes has been recognized as one of several strategies to curb tobacco consumption [2]. To control tobacco use, policymakers need to know the magnitude of price elasticity of demand for cigarettes. This information is important if increasing prices (i.e. through a tax on cigarettes) is used as a measure to control tobacco smoking while at the same time maximizing revenue. The importance of estimating elasticities of demand for cigarettes is therefore in its use for pricing and tax simulations. Not surprisingly, dozens studies that estimates price elasticities of demand for cigarettes have been done elsewhere [2,3].

Economists have increasingly examined addictive behaviors of smoking in theoretical and empirical economic models. A standard economic model assumes that consumers demand goods (including cigarettes and other tobacco products) in order to maximize their utility subject to a set of constraints such as prices, income, and other factors. This implies consumption decisions at a given point in time are independent of past choices [3]. Since cigarettes are highly addictive products (due to nicotine [4]), decisions regarding their consumption at any moment depend on previous choices. That is, a smoker who is addicted to a cigarette product must by definition have bought the product before and will require the same or larger quantities as before to maintain the addiction. Similarly, a smoker will suffer significant adjustment costs if consumption is stopped.

Chaloupka and Warner [2] have divided economic models of addiction into three groups: imperfectly rational, myopic, and rational. These models differ in the assumptions made about the extent of rationality among consumers of addictive products. The former models assume stable but inconsistent short-run and long-run preferences, i.e., individuals' preferences are not consistent over their life-cycle. In myopic models, individuals recognize the dependence of current addictive goods consumption on past consumption, but ignore the impact of current and past choices on future consumption decisions when they make current choices. Whilst in the rational addiction (henceforth RA) models, individuals incorporate the interdependence between past, current, and future consumption into their utility-maximization. This is in contrast to the assumption, implied in myopic models of addictive behavior, that future implications are ignored when making current decisions. In the RA models, individual recognizes the addictive nature of goods and decides to consume it because his/her pleasure gains are greater than the cost of the activity which includes health problems and lower utility later in life. The RA model [5] has become a standard approach in the analysis of addictive goods, and such model has been tested empirically to study numerous products, see Auld and Grootendorst [6] for reviews. Key important factor contributes to the popularity of the RA model is that such model has important policy implications, i.e., optimal taxation of addictive and harmful goods must include only the external costs that this behavior causes on other members of the society.

On this other hand, psychological studies of harmful addiction also have introduced three basic dimensions of: tolerance, withdrawal and reinforcement (positive effects of habits) that are now part of the formal economic models of addictive behavior [7]. Tolerance indicates that a given level of consumption is less satisfying when past consumption has been greater. In other word, a higher level of consumption is needed in the future to have the same utility for the given level of current consumption. Withdrawal denotes the loss of satisfaction following consumption cessation. For instance, smoker does not get any nicotine that produces unpleasant physiological symptoms. Whilst reinforcement means that greater current consumption of addictive good causes its future consumption to rise, e.g. smoking becomes an established habit. Thus, reinforcement dimension requires that past cigarette consumption motivate present consumption by increasing marginal utility derived from cigarettes more than the present value is reduced through the marginal harm from future consumption.

Addicts in public health point of view refer to psychological disorder, i.e. smokers acquire habit formation to the act of smoking, and physical addiction to nicotine. This view starts from the observation that smoking is bad for the smoker's health, and then goes on to conclude that individuals do not derive benefits from smoking. Whilst in economic sense, addicts is in fact a combination of habit and preference adaptation which starts with subjective individual preferences where individual's smokers reveal that they gain net utility (or satisfaction) from tobacco consumption. From the above perspectives, it implies that addicts in public health are among the addicts in the economist sense where addicts in the latter's construct picks out a larger dimension.

This study provides an empirical test of the RA of cigarette demand in Indonesia, a country that represents a significant contributor to the global burden of disease from tobacco-related illnesses. With the fourth largest population in the world, Indonesia in 2002 ranked as the fifth largest consumers of cigarettes (182 billion) behind China (1.7 trillion), USA (463 billion), Russia (375 billion), and Japan (299 billion) (Ahsan A, Wijono N: The impact analysis of higher cigarette price to employment in Indonesia, submitted). Cigarettes consumed increased from 33 billion in 1970 to 217 billion in 2004. Our study adds evidence to the existing empirical works for testing the RA hypothesis which yield mixed results, and are often described as less than convincing due to implausible discount rates, unsteady demand and low price elasticities [5,6,8-10]. Addiction in our study is a combination of habit and preference adaptation. For public health priorities, our model indicates that "addiction" can be conditioned and does respond to incentives. So for both revenue and health policymakers, we provide a methodological innovation for analyzing how tobacco tax policy can maximize revenue given public health goal or vice versa.

## Methods

### Model Specifications

The RA models assume that consumers take into account all future effects of their present consumption to maximize life-time utility or happiness [5]. Consumers realize the future harm and utility of their current consumption decisions. The quantity of current cigarettes consumption depends on past (lag) consumption, future (lead) consumption, prices of cigarettes, and other factors as follows:

(1) Cit=β0+β1Cit−1+β2Cit+1+β3Pcit+β4Pait+β5x′it++υi+di+εit

where *i *is an individual, *t *is time, *C *is consumption of cigarettes, *Pc *and *Pa *is the price of cigarettes and alcohol, respectively, *x' *is a vector of exogenous variable that affect consumption of cigarettes, υ*_i _*is individual fixed effects that control for the agent time invariant preferences and marginal utility of wealth, *d_t _*are time fixed effects to control unanticipated changes in wealth, and *ε_it _*is the error term.

Equation (1) allows for direct tests of addiction and rationality. The statistical significance of the coefficient of future consumption, *C*_*t*+1, _together with a reasonable estimate of the discount rate, gives a direct test of a RA model against an alternative model in which consumers are myopic addicts [5,9,10]. For addictive goods, equation (1) implies that current consumption is positively related to past consumption, with the degree of addiction reflected by *β*_1_. Similarly, given the assumption of rational behavior and the symmetry present in the model, future consumption (*β*_2_) has a positive impact on current consumption.

The ratio of the coefficient on the lead to that on the lags, *β_2_/β_1_*, gives an estimate of the discount factor [6]. The implied discount rate is computed using this expression (*β_1_/β_2 _*- 1). The effects of price on demand for cigarette can be obtained from the solution to the second-order difference equation (1). Coefficient estimate of the cigarette prices indicates the short run elasticity, whilst the long run elasticity is computed using the following expression: $\frac{\partial E(LnC_{it})}{\partial E(LnPc_{it})}=\frac{\hat{\beta_{3}}}{\left(1-\hat{\beta_{1}}-\hat{\beta_{2}u}\right)}$

### Estimators

Applying ordinary least squares (OLS) in equation (1) could lead to biased parameter estimates for two reasons. First, *ε_it _*may be serially correlated with and through suspected endogenous variable *C*_*it*-1 _and *C*_*it*+1_. Second, equation (1) was derived assuming perfect certainty on prices and other variables [5], and thus when unexpected changes in these variables causes individuals to revise their consumption plans at each time period. Thus, *C*_*it*+1 _should be seen as the planned future consumption, which may or may not be equal to the realized future consumption if there unexpected changes in period *t *+ 1, implying there is measurement error when we use actual values of *C*_*it*+1_.

Instrumental variables (IV) techniques are often used to estimate equation (1) due to endogenous of lagged and future consumption variables [6]. Here, we consider three estimators: two-stage least square (2SLS), Generalized Method of Moment (GMM) and system-GMM. The 2SLS is a two-step estimation procedure to correct endogeneity of the regressors. In particular, variables *C*_*it*-1 _and *C*_*it*+1 _are regressed in the first stage on known, observed personal characteristics (exogenous right hand side equation (1)) and the instruments *z_i_*, as follows:

(2) Cit*=β0+β1zi+β2Pcit+β3Pait+β4x′it+υi+dt+εit

where *C*_it* _are *C*_*it*-1 _and *C*_*it*+1; _*z_i _*are the potential instruments, and all else is as defined in equation (1). This stage generates predicted values for the endogenous proxy variables. The dependent variable is then regressed on all exogenous right hand side variables and the predicted values derived from the first stage regressions.

The 2SLS only assumes simultaneous exogeneity for *P_it _*and *x_it _*, and does not eliminate *υ_i _*in (1). Thus, the endogeneity of *C*_*it*-1 _and *C*_*it*+1 _are likely to be worse. A within transformation of equation (1) was done to eliminate *υ_i _*with the following transformation in first-differences [11]:

(3) ΔCit=β1ΔCit−1+β2ΔCit+1+β3ΔPit+β4Δx′it+Δdi+Δεit

where *t *= 3,...,*T*-1. Equation (3) allows one to apply instrumental variable techniques without assuming strict exogeneity of *P_it _*and ${x^{\prime }}_{it}$.

Our strategy is to find a set of instruments z*_it _*that are uncorrelated with Δ*ε_it _*and correlated with the regressors, and apply GMM to equation (3) using the orthogonality condition that E(*z_it_*, Δ*ε_it _*) = 0. The GMM estimator produces consistent estimate where hetersoskedasticity (or non-constant variance) arise, and asymptotically efficient estimates of the parameters of interest when the errors are serially independent [12]. The estimator does estimation on a set of orthogonal conditions which are the products of equations and instruments [13].

GMM and system-GMM are also used to deal with errors-in-variables and unobservable heterogeneity[11,14]. Given that GMM estimator with too many overidentifying restrictions perform poorly in finite samples [10,13], we applied the methods, developed by David Roodman [15] and implemented "xtabond2" module for STATA, of reducing the bias caused by too many overidentifying restrictions. A finite sample correction was applied to the robust two-step covariance matrix calculated for system-GMM estimator.

We utilized several diagnostic tests to evaluate the overall specifications of the models, and to select the most appropriate estimator. An endogeneity test was employed using the Hausman specification tests. If both *C*_*it*-1 _and *C*_*it*+1 _indeed exogenous, we would opt to OLS, otherwise either 2SLS, GMM or system-GMM call for. While to single out between 2SLS and GMM estimator, a various Pagan and Hall's (1983) test for heteroskedasticity was adopted [16]. Since we explored system-GMM, our selection doesn't end at this stage. Moreover, the consistency of the coefficient estimates of IV approach and the endogeneity test are dependent on the accuracy of instruments used. We employed several IV tests to evaluate whether there may be a bias from weak instruments and whether the instruments are orthogonal to the error process.

### Data and Variables

This study used individual aggregated data obtained from three-wave a panel of the Indonesian Family Life Survey (IFLS) data, conducted in 1993 (IFLS1), 1997 (IFLS2) and 2000 (IFLS3). Frankenberg and Karoly (1995), Frankenberg and Thomas (2001), and Straus *et al. *(2004) described more fully IFLS1, IFLS2 and IFLS3, respectively [17-19].

IFLS contains measures of smoking behavior from individuals aged 15 and above. Table 1 gives definition and descriptive statistics of the variable. We measure cigarette consumption (the dependent variable) as the number of cigarettes per day smoked as recalled by the individual at the time of the interview. The main explanatory variable is the number of cigarettes smoked as recorded in previous wave (*C*_*t*-1_) and in the next wave (*C*_*t*+1_) from the current interview. These variables measure the effects of past and future cigarette consumption on current marginal utility of cigarette consumption. We included measures of cigarette (*Pc_t_*) and alcohol (*Pa_t_*) prices at the time of the interview. Other time-varying explanatory variables included a monthly income proxy (from expenditure recall data), expressed as a real value with 2,000 CPI data. To obtain a per-equivalent adult measure of consumption, all income proxy data was adjusted for family size.

**Table 1:** Definition of variables used in the models and its descriptive statistic.

		IFLS 1993		IFLS 1997		IFLS 2000		Pooled	
					
Variable	Definition	Mean	**SD**.	Mean	**SD**.	Mean	SD	Mean	**SD**.
*C*_*t*0_	Current cigarette consumption (ln)	2.162	0.789	2.233	0.745	2.210	0.722	2.207	0.746
*C*_*t-*1_	One lag cigarette consumption (ln)	n.a	n.a	2.156	0.776	2.234	0.748	2.203	0.760
*C*_*t*+1_	One lead cigarette consumption (ln)	2.242	0.739	2.218	0.717	n.a	n.a	2.230	0.727
*Pc*_*t*0_	Current price cigarette (ln)	4.169	0.665	4.407	0.245	5.374	0.168	4.623	0.701
*Pc*_*t*-1_	One lag price cigarette (ln)	n.a	n.a	4.156	0.669	4.405	0.245	4.280	0.520
*Pc*_*t*+1_	One lead price cigarette (ln)	4.403	0.246	5.369	0.166	n.a	n.a	4.882	0.526
*Pa*_*t*0_	Current price alcohol (ln)	7.673	1.129	8.374	1.076	9.331	1.024	8.612	1.263
Ln-exp	Monthly per-capita income (ln)	10.629	0.863	11.079	0.812	11.891	0.848	11.156	1.004
Working	1 if working, 0 otherwise	0.611	0.488	0.549	0.498	0.592	0.491	0.582	0.493
Wall	1 if dwelling wall is brick, 0 otherwise	0.519	0.500	0.610	0.488	0.660	0.474	0.588	0.492
Floor	1 if dwelling floor is permanent, 0 otherwise	0.191	0.393	0.149	0.356	0.112	0.316	0.155	0.362
Hhown	1 if dwelling is owned, 0 otherwise	0.794	0.404	0.823	0.382	0.805	0.396	0.805	0.396
Moslem	1 if Moslem, 0 otherwise	0.858	0.349	0.877	0.328	0.882	0.322	0.871	0.335

Instrumental variable techniques can only be applied if one finds instruments that satisfy two requirements: they (the instruments) must be correlated with the endogenous variable(s) and are orthogonal [12]. Appropriate instrumental variables in our context will play an important role in determining past and future consumption (a potentially endogenous variable) but will not affect current consumption (the dependent variable) except through past and future consumption. Here, we proposed lagged and lead prices serving as instruments for past and future cigarette consumption. Following Grossman et al. (1998) [20], we included variable that measure some of the life cycle events (e.g., lagged and lead individuals working status) as the instruments. This variable affects utility, and therefore partially determines *ε_it_*. We avoided lagged values of cigarette consumption as instruments for lead consumption due to concerns about serial correlation in the errors. Other dummy variables, which we consider to be proxies for wealth or economic stability, were also included as potential instruments: dwelling walls are brick; dwelling floor is permanent; dwelling is owned (1/0); and individual's religion.

## Results

### Descriptive Statistics

Seventy-seven percent of male age above 15 years reported to have ever smoked habit in 1993, compared to 69 percent in 1997 and 70 percent in 2000 (Table 2). In all datasets, current smoking rates only slightly lower than ever-smoking rates, correspondence to a very small quitter rates, less than 5 percent. The majority of male smokers smoke cigarettes relative to other products. In IFLS 2000, about 93 percent of male smokers chose cigarettes compare to only 1 percent chewing tobacco.

**Table 2:** Descriptive statistics (mean) of smoking behavior in Indonesia.

	IFLS 1993			IFLS 1997			IFLS 2000			Pooled		
				
	Female	Male	Total	Female	Male	Total	Female	Male	Total	Female	Male	Total
Ever-had smoking habit	0.12	0.77	0.42	0.07	0.69	0.35	0.06	0.70	0.36	0.08	0.71	0.37
Still having smoking habit	0.10	0.70	0.38	0.06	0.64	0.33	0.05	0.65	0.34	0.07	0.66	0.34
Stop smoking	0.02	0.07	0.04	0.01	0.05	0.03	0.01	0.05	0.03	0.01	0.06	0.03
Cigarettes	0.38	0.81	0.74	0.41	0.90	0.85	0.48	0.93	0.89	0.42	0.89	0.84
Self-rolled cigarettes	0.12	0.28	0.26	0.07	0.17	0.16	0.06	0.12	0.11	0.09	0.18	0.17
Chew tobacco	0.55	0.02	0.10	0.55	0.01	0.07	0.49	0.01	0.05	0.53	0.01	0.07
Smoke a pipe	0.01	0.01	0.01	0.00	0.01	0.01	0.00	0.00	0.00	0.00	0.00	0.00
Starting smoke, age (yrs)	27.2	21.5	22.3	27.7	20.5	21.4	27.3	20.4	21.3	27.4	20.8	21.6
# cigarettes smoked/day	7.4	11.3	11.0	7.9	12.6	12.3	7.0	12.2	11.8	7.4	12.1	11.7

Smokers among female are rare. About 12 percent of female reported to have ever smoked habit in 1993 and decrease to 6 percent in 2000. In contrary to male, female prefer to chew tobacco, over 55%, than smoke cigarettes. On average, male started smoked earlier than female (21 vs. 27 years of aged). The figure is consistent in all data. Average number of cigarettes smoked per day is about 12. There are no significant changes in the number of cigarettes smoked between 1993, 1997 and 2000. But, male smoke approximately 30-40 percent higher in the average number of cigarettes smoked per day than female. Since female differ substantially in the frequency and amount of cigarette consumed from males, we perform our analysis separately for male and female.

### Model Selections

The endogeneity test, Durbin-Wu-Hausman and Wu-Hausman, for the entire samples was 3.5 and 6.9, respectively, with a *p*-value of 0.031 (Table 3), reject the null hypothesis of exogeneity. The suspected endogenous variable indeed endogenous (*p*-value for male and female sample was 0.06 and 0.04, respectively), suggesting OLS results in inconsistent parameter estimates.

**Table 3:** Summary statistics test for selecting the best estimator.

	All sample			Male			Female		
			
	Statistics:		*P*-val	Statistics:		*P*-val	Statistics:		*P*-val
**Endogeneity test:**									
a. Lagged and lead consumption									
Wu-Hausman	F(2,1775):	3.5	0.031	F(2,1711):	2.8	0.061	F(2,56):	3.32	0.043
Durbin-Wu-Hausman	Chi^2^(2):	6.9	0.031	Chi^2^(2):	5.6	0.061	Chi^2^(2):	6.79	0.034
b. Only lagged consumption									
Wu-Hausman	F(1,1776):	5.1	0.031	F(1,1712):	3.6	0.057	F(1,57):	0.00	0.983
Durbin-Wu-Hausman	Chi^2^(1):	5.1	0.031	Chi^2^(1):	3.6	0.056	Chi^2^(1):	0.00	0.982
c. Only lead consumption									
Wu-Hausman	F(1,1776):	0.2	0.637	F(1,1712):	0.2	0.636	F(1,57):	6.12	0.016
Durbin-Wu-Hausman	Chi^2^(1):	0.2	0.636	Chi^2^(1):	0.2	0.635	Chi^2^(1):	6.21	0.013
									

**Heteroskedasticity test(s):**									
Pagan-Hall general test	Chi^2^(11):	15.5	0.159	Chi^2^(11):	16.2	0.133	Chi^2^(11):	9.05	0.618
Pagan-Hall test w/assumed normality	Chi^2^(11):	34.1	0.000	Chi^2^(11):	36.7	0.000	Chi^2^(11):	7.69	0.741
White/Koenker nR2 test	Chi^2^(11):	16.4	0.128	Chi^2^(11):	17.0	0.107	Chi^2^(11):	17.00	0.108
Breusch-Pagan/Godfrey/Cook-Weisberg	Chi^2^(11):	36.9	0.000	Chi^2^(11):	39.8	0.000	Chi^2^(11):	13.44	0.265
									

**Overidentifying restrictions test:**									
Sargan N*R-sq (2SLS)	Chi^2^(6):	8.7	0.192	Chi^2^(6):	10.4	0.108	Chi^2^(6):	0.96	0.987
Basmann test (2SLS)	Chi^2^(6):	8.7	0.193	Chi^2^(6):	10.4	0.108	Chi^2^(6):	0.79	0.992
Hansen J (GMM)	Chi^2^(6):	8.0	0.237	Chi^2^(6):	9.8	0.132	Chi^2^(6):	1.07	0.983
Hansen J (GMM-system)	Chi^2^(33):	20.1	0.962	Chi^2^(33):	21.7	0.934	Chi^2^(33):	8.53	1.000

To discriminate either 2SLS or GMM, we utilize results of the Pagan and Hall's test for heteroskedasticity [16]. For female sample, none of the test rejected the null hypothesis of homoskedasticity, and thus at this stage the 2SLS estimator is preferred. Whilst for total and male samples, the heteroskedasticity test with assumed normality and the Breusch-Pagan/Godfrey/Cook-Weisberg test rejected the null hypothesis at the 1 percent level. This gives evidence for opting GMM instead of 2SLS estimators.

Selecting either GMM or system-GMM for male sample, and choosing either 2SLS or system-GMM for female sample are performed based on several instrumental variables tests. A reduced form regression of the lags and leads consumption (equation 2) on the full set of instruments was estimated. The results are presented in Table 4. We adopted several statistical criteria to investigate whether the instrument (i) correlate with the lagged and future consumption and (ii) are orthogonal to the errors process. The former implies the instruments must be relevant and valid.

**Table 4:** First-stage regression of the lags and leads consumption: OLS estimates.

	Total				Male				Female			
			
	Lag		Lead		Lag		Lead		Lag		Lead	
						
	**Coef**.	SE	**Coef**.	SE	**Coef**.	SE	**Coef**.	SE	**Coef**.	SE	**Coef**.	SE
*Pc_t0_*	-0.364^†^	0.075	-0.232^†^	0.069	-0.373^†^	0.076	-0.257^†^	0.068	0.232	0.521	0.328	0.510
*Pa _t0_*	0.252 ^†^	0.020	0.227^†^	0.018	0.257^†^	0.020	0.226^†^	0.018	0.144	0.123	0.216^‡^	0.121
Ln-exp	-0.011	0.023	0.018	0.021	-0.008	0.024	0.034	0.021	-0.041	0.136	-0.378^†^	0.133
*Pc_t-1_*	0.055*	0.027	-0.016	0.025	0.052^‡^	0.028	-0.030	0.025	-0.022	0.153	-0.007	0.150
*Pc_t+1_*	-0.081	0.118	-0.301^†^	0.108	-0.067	0.121	-0.288^†^	0.108	-0.534	0.663	-0.719	0.649
Working*_t+1_*	-0.063	0.047	0.153^†^	0.043	-0.057	0.048	0.148^†^	0.043	-0.37^‡^	0.198	-0.132	0.194
Working*_t-1_*	0.150^†^	0.053	0.085^‡^	0.049	0.113*	0.057	0.028	0.051	0.186	0.206	0.010	0.202
Wall	-0.121^†^	0.034	-0.041	0.031	-0.120^†^	0.034	-0.053^‡^	0.031	-0.396	0.246	-0.127	0.241
Floor	-0.239^†^	0.049	-0.079^‡^	0.045	-0.244^†^	0.050	-0.086*	0.045	-0.114	0.338	-0.479	0.331
Hhown	-0.050	0.039	-0.038	0.035	-0.040	0.039	-0.016	0.035	-0.356	0.276	-0.735^†^	0.270
Moslem	-0.193^†^	0.053	-0.137^†^	0.048	-0.198^†^	0.053	-0.117^†^	0.048	-0.028	0.356	-0.818*	0.349
Constant	2.261^†^	0.550	2.925^†^	0.504	2.197^†^	0.565	2.904^†^	0.506	3.883	2.876	8.286^†^	2.816
												

# N	1783		1783		1719		1719		64		64	
# regressors K	6		6		6		6		6		6	
# instrument L	12		12		12		12		12		12	
# excl. instrum.	8		8		8		8		8		8	
*R*^2^	0.133		0.124		0.137		0.130		0.191		0.361	
Shea Partial *R*^2^	0.031		0.021		0.033		0.020		0.112		0.176	
Partial *R*^2^	0.036		0.024		0.034		0.021		0.16		0.253	
F-test of:												
- all instrument	24.77 ^†^		22.69 ^†^		24.58 ^†^		23.16 ^†^		0.37		2.67 ^†^	
- excl. instrument	8.22 ^†^		5.43 ^†^		7.53 ^†^		4.54 ^†^		1.24		2.2 *	

^‡^significant at 10%; *significant at 5%; ^†^significant at 1%; SE is robust standard errors

The relevancy of instruments was investigated by evaluating *R*^2 ^in the first-stage regression of equation (2) [21]. Our *R*^2 ^reveals the models explained a relatively high proportion of the variation for the suspected endogenous. For the entire samples, *R*^2 ^for the lagged and future consumption was 13% and 12%, respectively. As our models have two suspected endogenous variables, relying only on *R*^2 ^and *F*-test may not be enough to detect the relevance of the instruments. Hence, we used a Shea partial *R*^2 ^measure, which takes into account the inter-correlations among the instruments [22,23]. Table 4 also reports both Partial *R*^2 ^and Shea Partial *R*^2^. An estimated equation that yields a large value of the Partial *R*^2 ^and a small value of the Shea measure indicating the instruments lack sufficient relevance to explain all the endogenous regressors, and the model may be essentially unidentified [21]. With the exception of female sample, a gap between Partial *R*^2 ^and Shea partial *R*^2 ^is considerably low, and thus our models are well-identified.

The relevance of the instruments was also investigated using *F*-test to determine whether they correlated with the potentially endogenous regressors [24]. The null hypothesis of the *F*-test that the parameters of the covariates are jointly equal to zero was rejected, indicating the instruments are jointly significant. However, values of the *F*-test gives little doubt on the relevance of the instruments. A conservative rule of thumb for a single endogenous regressor is that the *F*-test less than 10 is an indicator of a weak instrument [21]. In our full sample (see last row of Table 4), *F*-test all instruments for the lagged and lead consumption were 25 and 23, respectively, whilst *F*-test excluded instrument *F*-(8; 1176) were 8 and 5 for the lagged and lead consumption, respectively. For female sample, results of the *F*-test even yielded worse performance, less than 2.

The validity of the instruments was tested by over-identification restrictions test. A Hansen's *J*-statistic, and both Sargan's and Basmaan's statistic tests were used in the case of GMM and 2SLS respectively (Table 3). The joint null hypothesis of these tests is that the excluded instruments are valid instruments (i.e. uncorrelated with the error terms), and that they are correctly excluded from the estimated equation. The test is distributed as a χ2 with degrees of freedom equal to the number of exceeding instruments. The critical value of the χ2 at the 95% level of significance with 6 degrees of freedom is 8, we therefore cannot reject the null of no overidentification. This suggests that the models are reasonably well specified and the instruments are valid.

Finally, the instruments are not only required to be correlated with the endogenous variable(s), they will also need to satisfy an orthogonal requirement [12], i.e. the z should be exogenous. We tested a subset of instruments using *C*-statistic test. that allowed us to test a subset of the original set for orthogonality conditions. Unfortunately, orthogonality requirements of the instruments cannot be satisfied. The *C*-statistics test, except for female, could not reject the null hypothesis of exogeneity. This implies that our subset instruments are endogenous. Both *C*-statistics test and *F*-test give more evidence for not using GMM estimator.

### Estimation Results

Results for cigarette demand equation (1) are shown in Table 5. The last row of the Table presents price elasticities of demand, discount factor and discount rate of time preference.

**Table 5:** Rational addiction cigarette consumption estimates: model comparisons.

	Total				Male				Female			
			
	OLS	2SLS	GMM	System GMM	OLS	2SLS	GMM	System GMM	OLS	2SLS	GMM	System GMM
*C_t-1_*	0.244^†^	0.520^†^	0.521^†^	0.846^†^	0.238^†^	0.485^†^	0.487^†^	0.790^†^	0.415^†^	0.114	0.292	1.096^†^
	[0.021]	[0.113]	[0.112]	[0.130]	[0.022]	[0.110]	[0.110]	[0.126]	[0.117]	[0.517]	[0.449]	[0.382]
*C_t+1_*	0.318^†^	0.133	0.119	-0.749^†^	0.315^†^	0.137	0.123	-0.761^†^	0.293^†^	0.866 *	0.806 *	-0.243
	[0.027]	[0.152]	[0.145]	[0.078]	[0.028]	[0.151]	[0.150]	[0.080]	[0.104]	[0.386]	[0.365]	[0.230]
*Pc_t0_*	-0.154^†^	-0.118^‡^	-0.129^‡^	-0.358^†^	-0.151^†^	-0.127^‡^	-0.138^‡^	-0.384^†^	-0.362	-0.367	-0.289	-0.567
	[0.045]	[0.070]	[0.066]	[0.084]	[0.046]	[0.073]	[0.072]	[0.087]	[0.261]	[0.337]	[0.320]	[0.575]
*Pa_t0_*	0.182^†^	0.152^†^	0.157^†^	0.253^†^	0.184^†^	0.159^†^	0.163^†^	0.234^†^	0.158	0.019	-0.006	0.448^‡^
	[0.019]	[0.035]	[0.035]	[0.060]	[0.019]	[0.037]	[0.037]	[0.062]	[0.110]	[0.139]	[0.133]	[0.225]
Ln-exp	-0.021	-0.016	-0.015	0.023	-0.027	-0.019	-0.017	0.03	0.131	0.286^‡^	0.294^‡^	0.037
	[0.018]	[0.018]	[0.018]	[0.056]	[0.018]	[0.019]	[0.019]	[0.058]	[0.098]	[0.155]	[0.154]	[0.309]
Constant	0.469*	0.333	0.36	1.455^‡^	0.527*	0.422	0.44	1.832*	-0.528	-1.489	-1.966	-1.494
	[0.226]	[0.351]	[0.354]	[0.768]	[0.231]	[0.374]	[0.372]	[0.776]	[1.327]	[1.848]	[1.764]	[2.369]
												

N	1790	1783	1783	1783	1725	1719	1719	1719	65	64	64	64
R-squared	0.41	0.33	0.33		0.40	0.34	0.34		0.45	0.25	0.27	
F-test	181.45^†^	104.72^†^	81.72^†^	27.63^†^	171.7^†^	79.72^†^	80.96^†^	26.34^†^	9.9^†^	5.06^†^	5.76^†^	2.51^‡^
# instruments		12	12	39		12	12	39		12	12	39
# excl. instruments		8	8			8	8			8	8	

Short-run elasticity	-0.154	-0.118	-0.129	-0.358	-0.151	-0.127	-0.138	-0.384	-0.362	-0.367	-0.289	-0.567
Long-run elasticity	-0.352	-0.340	-0.358	-0.396	-0.338	-0.336	-0.354	-0.395	-1.240	-18.35	2.949	-3.857
Discount factor	1.303	0.256	0.228	-0.885	1.324	0.282	0.253	-0.963	0.706	7.596	2.760	-0223
Discount rate	-0.233	2.910	3.378	-2.130	-0.244	2.540	2.959	-2.038	0.416	-0.868	-0.638	-5.510

^‡^significant at 10%; *significant at 5%; ^†^significant at 1%; Robust standard errors in [brackets]

Note: See equation (1): The short run price elasticity is the coefficients estimates of cigarette price, *β_3_*; the long run price elasticity is calculated using the expression $\partial E(LnC_{it})/\partial E(LnPc_{it})={\hat{\beta }}_{3}/(1-{\hat{\beta }}_{1}-{\hat{\beta }}_{2})$; and the implied discount factor is *β_2_/β_1 _*and the implied discount rate is *β_1_/β_2_*-1

Pooled OLS with robust cluster standard errors, for the entire sample as well as for male and female separately are reported in Table 5. OLS ignores the endogeneity of the lags and leads consumption. For the full sample, coefficient estimates of the leads and lags consumption are significant, and hence rejecting the myopic model in favor of future looking consumers. The future consumption term coefficient is higher than the lagged one giving rise to a negative discount rate. Price of cigarettes (negative) and alcohol (positive) were significant, but income was not. The findings on prices are the same when applied to male and female sample. While the signs and the significance levels of the coefficients are the same, the magnitudes are different. The short run price elasticity of demand for males and females, for instance, was estimated to be -0.15 and -0.36 respectively. The demand to be slightly higher price sensitive in the long run, with an estimated elasticity of -0.34 and -1.24 for males and females, respectively.

Table 5 also reports the 2SLS and GMM regression allowing for the endogeneity of lead and lagged consumption. The instruments used are those described in data and variables section. All standard errors are heteroskedastic consistent. Both 2SLS and GMM produces statistically significant results for variables lagged consumption, cigarettes price and alcohol price. One can observe that the magnitudes of prices variables are almost similar from both 2SLS and GMM estimates. The patterns hold true for the full sample, as well as for male and female samples. For males, the coefficient of lead consumption has a positive sign and is smaller than the coefficients of lagged one. This finding is consistent with the theory, which rises to a positive rate and reasonable time preference. Since it is insignificant, however, the RA hypothesis is rejected in favor of the myopic one. The estimated short run prices elasticity of cigarettes for male is -0.13 (in 2SLS) and -0.14 (in GMM), and the long run prices elasticity become triple, -0.34 (2SLS) and -0.36 (GMM). For female, we find demand to be substantially price sensitive in the long run, with an estimated elasticity of -18.3 (2SLS) and -2.95(GMM), compared to the short-run price elasticity which is only -0.37 (2SLS) and -0.29 (GMM).

Our primary results are displayed in Table 5, labeled system-GMM. This estimator increases the number of valid instruments, 38 in system-GMM compared with only 12 in both 2SLS and GMM estimators. For the total and male samples, all parameters estimates produced by a system-GMM, except monthly per-capita income, were statistically significant at the 1 percent level. Whilst for female sample, only the lagged consumption (*p*-value < 1%) and alcohol price (*p*-value < 10%) turned out to be statistically significant. A positive value of the lagged consumption (*p*-value < 1%) suggests that the effect of dependence, reinforcement and tolerance is significant. However, the lead consumption coefficient term turned out to be a negative, suggesting the RA model is rejected in favor of the myopic one. For male sample, the short and long run price elasticity of cigarette was estimated to be -0.38 and -0.39, respectively, and is significant at the 1% percent level, while for female, it was estimated to be -0.57 and -3.89 for short and long run elasticity, respectively.

## Discussion

This study tests the RA hypothesis using OLS, 2SLS, GMM, and system-GMM estimators. The former estimator produces consistent estimates of the coefficients and of their standard errors only when the regressors are exogenous and the error term is homoskedastic and serially uncorrelated [25]. The three later estimators allow one to control endogeneity of the regressors [14,26], but they are generally less efficient than OLS. Thus, there is a trade-off between loss of precision and having biased parameter estimates. Since arriving at the choice of most appropriate model is a difficult process but not often documented in the literature in great details, we describe a series of criteria that helped us selecting the most appropriate econometric technique in such a case.

We find the evidence for endogeneity of both lagged and future consumption. This led us to apply the methods that treat regressor as endogenous. While 2SLS has been applied widely to test the RA model [6,27-30], among other to correct the endogeneity problem, we leave such estimator out for two reasons. First, unknown hetersoskedasticity exist, especially in the total and male samples, and this lead to give an invalid inference since the standard error is inconsistent [23]. Second, results of the instrumental variable tests indicating little doubt of the excellence our instruments, in particular for female sample where the *F*-test less than 10 [21]. The later raises concern on the use of GMM estimator as well. Thus, we conclude that system-GMM estimator is probably the best to estimate our dynamic specifications model.

The RA hypothesis is accepted when the coefficient of the future consumption is a positive and significant, and when the discount rate has a reasonable value [5,9,31]. Our finding rejects the RA hypothesis in favor of myopic one. Estimates derived from a system-GMM yielded a significant negative value of the coefficient associated to the future consumption, and therefore the estimated value of the discount factor was implausible.

A rational smoker engages in a rational learning process, balances the utility value of smoking with expected utility loss and selects the efficient risk level [3]. This behavior doesn't hold true for Indonesian smokers. They neglect future consequences of smoking risks, and ignore the impact of current and past choices on future consumption decisions when making current choices. This may due to smokers underestimate tobacco's danger relative to other health risks, and they fail to fully internalize these risks. Unfortunately, we are unable to test this assumption within our models and empirical specifications.

Nevertheless, the findings that Indonesian smokers are irrational, e.g. they are myopic addicts, are an important message for public health policy. Anecdotal, tobacco industries in the county provide more attractive and thorough advertisement as well as provide many favorable messages about smoking on their products than public health campaign do. If we believed that smokers are misinformed about a key risk of smoking, our findings would imply that policy makers may need strategies that would change smokers' perceptions about a key risk of smoking. In light of this view, the promotion of more informed and responsible smoking should become policy objective. Policy makers have to redesign current public health campaign against cigarette smoking in the country.

Another important finding from this analysis is very interesting. Cigarette consumption is found to be negatively related to price, and the long-run cigarette price effects (or equilibrium multiplier) exceeded the short-run effects. From a public health perspective, these findings are of substantial interest, suggesting an increase in the price of cigarettes via excise taxes could lead to a significant fall in cigarette consumption in the long-run. Future increase in the tax will reduce the number of ex-smokers returning to cigarettes and will reduce consumption among continuing smokers. They also will induce some smokers to quit and prevent others from becoming regular or persistent smokers [2]. Empirical evidence from South Africa shows that a doubling of the real price of cigarettes between 1993 and 2003 would reduce consumption by a quarter in the short term [32]. These gains would be significant in South Africa or any other country struggling with the public health consequences of high rates of tobacco consumption like in Indonesia.

Our study also finds the long-run cigarette price effects for female are greater than one, in absolute value, implying the demand to be more elastic in the long-run than in the short-run. A long-run price elasticity of -3.85 among female smokers indicates that increasing cigarette prices via excise taxes can be an effective tool to reduce cigarette consumption for this population. However, since the demand for cigarettes is more elastic in the long-run, further excise tax increases are more likely to act as a tobacco control mechanism in the long-run rather than as a constant source of government revenue. In such a case, price increases brought about by higher taxes would cause government revenue to decrease as the proportionate change in prices would lower the proportionate change in consumption.

The short-run elasticity of cigarette demand gives the percentage variation in the consumption of cigarette in the first year after a permanent change in the current price and all future prices, with past consumption held constant. Whilst Hu and Mao [33] argue that cigarettes price elasticities are higher in developing countries than in developed countries due to the relatively low incomes level in developing countries that makes people react more sensitively to price changes, the short-run price estimates in our study are quite comparable to other developed countries, range between -0.25 to -0.5 [2]. Our short-run price estimates coincides with the previous study in the country by Djutaharta et al. (2005) who found a ten percent increase in cigarette prices lowered the demand by 3.4 percent [34].

## Conclusions

This study estimates the demand for cigarette in Indonesia according to the rational addiction framework. The demand equation is tested on individuals aggregated data taken from three-wave a panel of the Indonesian Family Life Survey covering the periods 1993-2000. We explore several estimators, and select the best alternative one to overcome the econometric problems faced in presence of endogenous or predetermined variable. Findings confirm that while the effect of dependency, reinforcement and tolerance is significant (and hence cigarette is an addictive good), the rational addiction hypothesis is rejected in favor of myopic one. This finding calls health policymakers to redesign current public health campaign against cigarette smoking. We also find demand to be more price sensitive in the long-run (and female) than the short-run (and male), suggesting an increase in the price of cigarettes could lead to a significant fall in cigarette consumption in the long-run. The short-run cigarette price elasticity for male and female is estimated to be-0.38 and -0.57, respectively, and the long-run one is -0.4 and -3.85, respectively.

## Competing interests

The authors declare that they have no competing interests.

## Authors' contributions

BH participated in the design of the study, managed the data, performed the statistical analysis, interpretation of the results, drafted the manuscript and revised it critically for important intellectual content. HT contributed to conception of this study, helped to draft the manuscript and revised it. All authors read and approved the final manuscript.
